# Pituitary Crooke Cell Adenoma: Two Cases of an Aggressive Pituitary Adenoma

**DOI:** 10.1210/jcemcr/luad114

**Published:** 2023-11-16

**Authors:** Adeyinka Taiwo, Vijayvardhan Kamalumpundi, Nicole Becker, Marcelo Correia

**Affiliations:** Division of Endocrinology, Department of Internal Medicine, University of Iowa Health Care, Iowa City, IA 52246, USA; Roy J. and Lucille A. Carver College of Medicine, University of Iowa, Iowa City, IA 52246, USA; Roy J. and Lucille A. Carver College of Medicine, University of Iowa, Iowa City, IA 52246, USA; Roy J. and Lucille A. Carver College of Medicine, University of Iowa, Iowa City, IA 52246, USA; Department of Pathology, University of Iowa Health Care, Iowa City, IA 52246, USA; Division of Endocrinology, Department of Internal Medicine, University of Iowa Health Care, Iowa City, IA 52246, USA; Roy J. and Lucille A. Carver College of Medicine, University of Iowa, Iowa City, IA 52246, USA

**Keywords:** Pituitary adenoma, ACTH, Crooke cell, Cushing disease, Hyalinization

## Abstract

Crooke cell adenoma (CCA) is a rare and aggressive subtype of a corticotroph adenoma, which requires lifetime surveillance. There have been 106 cases of CCAs reported in the English literature. We describe 2 cases of CCA, a 48-year-old man and an 84-year-old woman who both presented with binocular diplopia and temple pain. Neither case had clinical Cushing syndrome. Laboratory values for the 48-year-old man revealed, adrenocorticotropin (ACTH) 103 pg/mL (22 pmol/L) (RR: 7-63 pg/mL) and evening cortisol 14 µg/dL (386 nmol/L) (RR: 2.7-10.5 µg/dL). Computed tomography imaging demonstrated a mass adjacent to the right cavernous sinus extending into the sphenoid sinus. He underwent tumor resection with adjuvant radiation and has had a stable residual tumor for 4 years. Preoperative laboratory values for the 84-year-old woman revealed, ACTH 69 pg/mL (15 pmol/L) (RR: 7-63 pg/mL) and evening cortisol 16.2 µg/dL (447 nmol/L) (RR: 2.7-10.5 µg/dL). Brain magnetic resonance imaging revealed, a mass compressing the optic chiasm. She underwent resection and has had a stable residual tumor for 2 years. Surgical pathology in both cases revealed cytoplasmic hyaline deposits of more than 50% of the tumor cells, consistent with CCA. The CCA although rare, should be considered when evaluating cases with subclinical Cushing disease and visual symptoms.

## Introduction

Crooke cell adenomas (CCAs) are a rare subtype of pituitary tumor accounting for less than 1% of pituitary adenomas [1]. CCAs usually present as an invasive macroadenoma that may produce adrenocorticotropin (ACTH) or remain clinically nonfunctional [2]. They are typically detected on brain imaging in patients with headaches or visual disturbances. Cases can also present with Cushing syndrome, though this may not always be true in cases of minimal hormonal hypersecretion. CCAs exhibit a strong female predominance in 72.8% of reported cases and have a predilection for those in the late fourth decade of life [3]. Compared to ACTH-producing adenomas, CCAs exhibit more aggressive behavior, can invade the cavernous and sphenoid sinuses, and though rare may cause central nervous system and systemic metastasis [3].

CCAs are a subtype of corticotroph adenoma with more than half of tumor cells containing pale eosinophilic hyaline deposits in the cytoplasm [1]. The cytoplasmic hyaline material identified on hematoxylin and eosin stain is composed of cytokeratin filaments accumulating around the nucleus. The hyaline change is reported to be the result of excess glucocorticoid exposure [4]. The neoplastic Crooke cells demonstrate nuclear immunoreactivity for T-box transcription factor (T-PIT) and variable ACTH immunoreactivity restricted to the periphery of the cytoplasm. The keratin filaments of the Crooke hyaline can be highlighted with cytokeratin immunostains like CK8 and CK18.

To date, 106 cases of CCA have been described in the literature in English, and the prognosis of these cases has not been well characterized [3]. Herein, we present the radiologic, histologic characteristics, and clinical outcomes in 2 cases with CCA.

## Case Presentation

### Case 1

A 48-year-old man presented to the emergency department with acute right eye pain and right temple pain. He was not having any vision changes, light sensitivity, associated nausea or vomiting, and had no aura. The neurological examination was unremarkable. He was given a dose of sumatriptan for presumed atypical migraine with symptom resolution and was sent home with an as-needed prescription of sumatriptan. Our case again presented 5 days later to the emergency department with acute right eye pain, right temple pain refractory to sumatriptan, and a new symptom of binocular diplopia. Physical examination revealed, a severe sixth cranial nerve palsy, bilateral optic disc pallor, and a mild right superotemporal visual field defect. He did not have clinical signs of Cushing syndrome such as moon facies, dorsocervical or supraclavicular fat pad, or purple striae. His past medical history was significant for type 2 diabetes, traumatic brain injury with resultant palsy of the right oculomotor and facial nerve 25 years ago, cerebrospinal fluid leak with bacterial meningitis 11 years prior, and left cochlear implant.

### Case 2

An 84-year-old woman presented to the emergency department after a 2-week history of right-sided ptosis and binocular diplopia. Her physical examination revealed a down and abducted position of the right eye with a dilated pupil, consistent with right third cranial nerve palsy. Left eye extraocular motility was normal. She did not have any clinical features of Cushing syndrome. She had a past medical history of type 2 diabetes and glaucoma.

## Diagnostic Assessment

### Case 1

**Figure 1.**
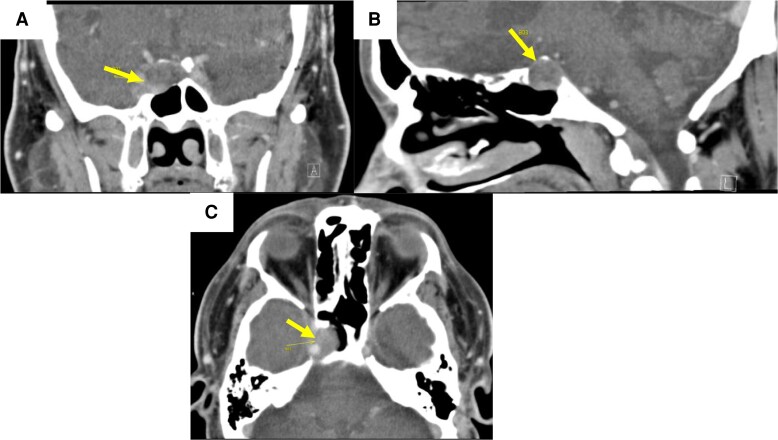
Computed tomography scan of the brain from case 1. Arrows are pointing to the mass in all images. A, Coronal section of the sellar mass medial to the right cavernous sinus. B, Sagittal scan showing the mass extending into the sphenoid sinus. C, Axial section showing mild contrast enhancement of the mass abutting the superior orbital fissure.

Head computed tomography (CT) with contrast demonstrated a 1.9 × 2.1 × 1.5 cm soft tissue–density mass medial to the right cavernous sinus and carotid artery extending into the sphenoid sinus. There was an expansile bony change to the floor and right dorsum of the sella. The mass showed mild contrast enhancement and abutted the superior orbital fissure (Fig. 1). Magnetic resonance imaging (MRI) of the brain was not obtained due to history of the left cochlear implant. Preoperative pituitary function tests revealed elevated ACTH 103 pg/mL (22 pmol/L) (reference range [RR]: 7-63 pg/mL), and normal random plasma cortisol 14 µg/dL (386 nmol/L) (RR: 2.7-10.5 µg/dL) consistent with subclinical Cushing disease. Pituitary function tests were otherwise unremarkable (Table 1).

**Table 1.** Hormone laboratory values preoperatively, postoperatively, 1, and 2-year follow-up

Hormone	ACTH	TSH	LH	FSH	IGF-1	PRL	Free T4	Evening cortisol	Total testosterone^a^
Reference range (SI Unit)	7-63 pg/mL (1.5-13.7 pmol/L)	0.4-4 µIU/mL (0.4-4 mIU/L)	1.7-8.6 mIU/mL (1.7-8.6 IU/L)	1.5-12.4 mIU/mL (1.5-12.4 IU/L)	69-224 ng/mL (9-29.3 nmol/L)	4-15.2 ng/mL (4-15.2 µg/mL)	0.8-1.8 ng/dL (10.3-23.2 pmol/L)	2.7-10.5 µg/dL (74.5-289.7 nmol/L)	249-836 ng/dL (8.6-29 nmol/L)
**Time Point**									
**Case 1**									
Preop	**103 pg/mL (22.7 pmol/L)**	1.10 µIU/mL (1.10 mIU/L)	3.8 mIU/mL (3.8 IU/L)	2.7 mIU/mL (2.7 IU/L)	208 ng/mL (27.3 nmol/L)	**17.6 ng/mL (17.6 µg/mL)**	**0.67 ng/mL (8.6 pmol/L)**	**14 µg/dL (386 nmol/L)**	**175 ng/dL (6 nmol/L)**
Postop	–	–	–	–	–	–	–	8.3 µg/dL (229 nmol/L)	283 ng/dL (9.8 nmol/L)
1 y	26 pg/mL (5.7 pmol/L)	–	–	–	–	–	–	5.3 µg/dL (146 nmol/L)	258 ng/dL (9 nmol/L)
2 y	28 pg/mL (6.2 pmol/L)	2.49 µIU/mL (2.49 mIU/L)	2.5 mIU/mL (2.5 IU/L)	2.6 mIU/mL (2.6 IU/L)	103 ng/mL (13.5 nmol/L)	**25.5 ng/mL (25.5 µg/mL)**	0.7 ng/mL (9 pmol/L)	4 µg/dL (110 nmol/L)	**198 ng/dL (6.9 nmol/L)**
**Case 2**									
Preop	**69 pg/mL (15.2 pmol/L)**	**0.29 µIU/mL (0.29 mIU/L)**	**0.4 mIU/mL (3.8 IU/L)**	2.4 mIU/mL (2.4 IU/L)	83 ng/mL (10.9 nmol/L)	31.3 ng/mL (31.3 µg/mL)	0.84 ng/mL (10.8 pmol/L)	**16.2 µg/dL (447 nmol/L)**	8 ng/dL (0.28 nmol/L)
Postop	17 pg/mL (3.74 pmol/L)	–	–	–	–	–	0.86 ng/mL (11.1 pmol/L)	–	–
1 y	20 pg/mL (4.4 pmol/L)	2.55 µIU/mL (2.55 mIU/L)	–	–	–	–	0.98 ng/mL (12.6 pmol/L)	9.9 µg/dL (273 nmol/L)	–
2 y	19 pg/mL (4.2 pmol/L)	2.55 µIU/mL (2.55 mIU/L)	–	–	–	–	0.98 ng/mL (12.6 pmol/L)	**12.8 µg/dL (353 nmol/L)**	–

Abnormal values are shown in bold font. Values in parenthesis are International System of Units (SI).

Abbreviations: ACTH, adrenocorticotropin; FSH, follicle-stimulating hormone; IGF-1, insulin-like growth factor 1; LH, luteinizing hormone; PRL, prolactin; T4, thyroxine; TSH, thyrotropin.

^*a*^Testosterone ranges for males 19 to 49 years is 249 to 836 ng/dL (8.6-29 nmol/L) and females older than 50 years is 2 to 41 ng/dL (.07-1.4 nmol/L).

### Case 2

MRI of the brain with and without contrast revealed, a 2.0 × 1.8 × 1.7 cm partially cystic sellar/suprasellar mass compressing the optic chiasm with fluid levels seen within the lesion (Fig. 3). The lesion extended into the right cavernous sinus and abutted the medial aspect of the carotid artery. Pituitary function tests revealed elevated ACTH 69 pg/mL (15 pmol/L) (RR: 7-63 pg/mL) and elevated evening cortisol 16.2 µg/dL (447 nmol/L) (RR: 2.7-10.5 µg/dL), consistent with subclinical Cushing syndrome. Other pituitary function tests were unremarkable (see Table 1).

**Figure 2.**
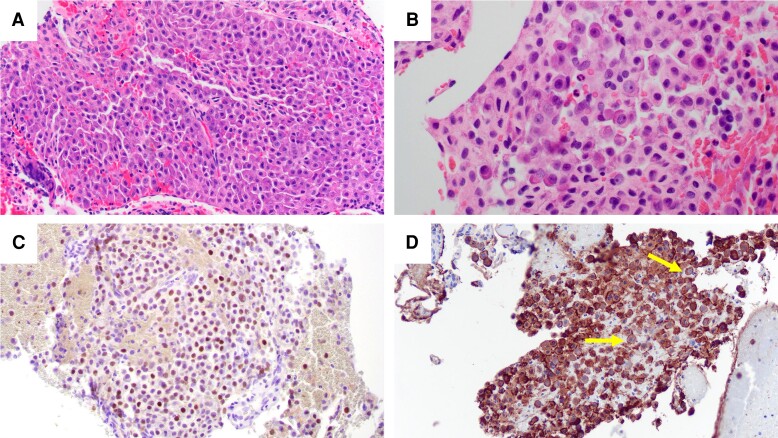
Surgical pathology examination and immunohistochemistry of the sellar mass from case 1. A, Hematoxylin and eosin stain of the pituitary adenoma, 200×. B, Many cells contain Crooke's hyaline around the nucleus, 400×. C, T-PIT transcription factor immunohistochemical stain confirms corticotroph lineage, 200×. D, Adrenocorticotropin (ACTH) immunohistochemical stain showing many cells are positive for ACTH and have peripheral accentuation of the stain with negativity around the nucleus supporting the presence of Crooke's hyaline (arrows), 200×.

**Figure 3.**
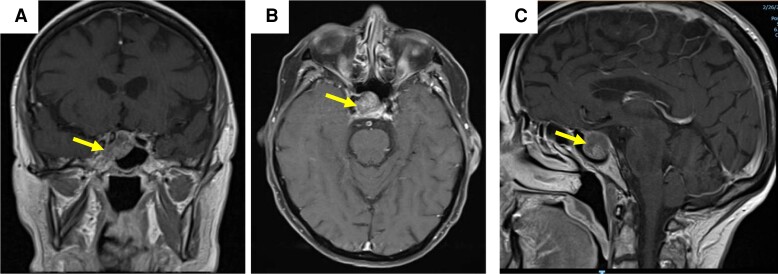
T1-weighted magnetic resonance imaging of the brain from case 2. Arrows point to the mass in all images. A, Coronal section of partially cystic sellar and suprasellar mass causing mass effect on the optic chiasm. The mass is medial to the right cavernous sinus. B, Sagittal cut showing the mass abutting the optic chiasm in the sella. C, Axial cut showing a well circumscribed mass in the sella.

## Treatment

### Case 1

The case 1 underwent endoscopic transsphenoidal resection of the pituitary mass. Pathologic examination revealed a cellular tumor composed of sheets of neuroendocrine tumor cells. The usual granular basophilic cytoplasm of the anterior pituitary cells was displaced by homogeneous eosinophilic material (Figs. 2A and 2B). The eosinophilic material was present in more than 50% of the tumor cells. Mitotic figures were not identified. The tumor cells were positive for T-PIT immunostain (Fig. 2C) and negative for pituitary transcription factor 1 (PIT1; not pictured). ACTH immunohistochemical staining showed that the hormone expression was confined to the periphery of the cell while the paranuclear region was negative (Fig. 2D). The tumor cells were negative for prolactin and growth hormone. Therefore, the tumor was diagnosed as a corticotroph pituitary adenoma, consistent with CCA. Postoperatively, he received 54 Gy in 30 fractions to the sella because of the high risk of recurrence and was placed on a short hydrocortisone taper.

### Case 2

**Figure 4.**
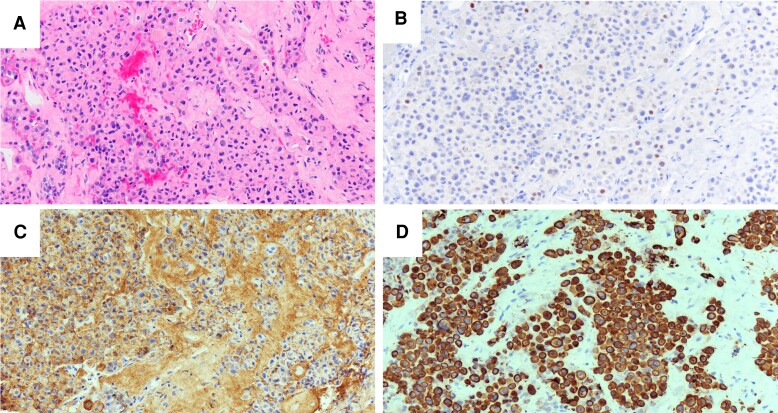
Surgical pathology examination and immunohistochemistry of the sellar mass from case 2. A, Hematoxylin and eosin stain of the pituitary adenoma with most cells containing Crooke's hyaline, 200×. B, T-PIT transcription factor immunohistochemical stain confirms corticotroph lineage, 200×. C, Adrenocorticotropin (ACTH) immunohistochemical stain demonstrates most cells are negative for ACTH in the cytoplasm but positive at the cell membranes, 200×. D. Pankeratin immunohistochemical stain demonstrating concentric paranuclear accentuation in the tumor cells in the presence of Crooke's hyaline, 200×.

The case 2 underwent a subtotal transsphenoidal resection with no complications. Microscopic examination of the tumor revealed sheets of neuroendocrine tumor cells, with greater than 50% of cells containing pale eosinophilic paranuclear hyaline material suggestive of Crooke hyaline (Fig. 4A). The tumor cells were positive for T-PIT and negative for pituitary transcription factor 1, supporting the diagnosis of a corticotroph pituitary adenoma (Fig. 4B). Immunohistochemical staining with ACTH (Fig. 4C) and pankeratin (Fig. 4D) demonstrated the ACTH was positive only at the cell periphery and negative around the nucleus. The pankeratin was positive concentrically, supporting the presence of Crooke hyaline. Taken together, these findings supported the diagnosis of a CCA. She was treated postoperatively with a short hydrocortisone taper. She did not receive adjuvant radiation therapy due to her advanced age.

## Outcome and Follow-up

### Case 1

The right eye pain and abduction deficit improved, and diplopia returned to baseline. Two, 6, and 12-month follow-up head CTs revealed stable, small residual enhancing tissue. Pituitary function tests and 24-hour urine cortisol conducted 24 months post surgery were all normal. Therefore, no hormone replacement was needed. He had a residual mild bilateral hemianopsia and a partial third nerve palsy. He has remained clinically stable 4 years post surgery with most recent imaging demonstrating unchanged residual tumor in the right cavernous sinus encasing the right internal carotid artery.

### Case 2

Postoperatively, she had improvement of her right third cranial nerve palsy, albeit with continued mild palsy and diplopia. Postoperative laboratory results revealed ACTH 17 pg/mL (4 pmol/L) (RR: 7-63 pg/mL). Postoperative cortisol levels were not drawn. The 4-month follow-up brain MRI demonstrated a decrease in size of the residual tumor (1.0 × 1.0 × 1.0 cm) with less shift of the pituitary stalk and compression of the right cavernous sinus. She had mild residual right third cranial nerve dysfunction with anisocoria and ptosis. She has remained clinically stable 2 years post surgery, with the most recent imaging demonstrating a small residual hypoenhancing lesion along the right surgical margin but no new growth. Other pituitary laboratory results were unremarkable (see Table 1).

## Discussion

We report 2 cases who presented with rapid onset of visual defects and headache. Head imaging revealed sellar masses, necessitating surgical removal. Ultimately, both cases were diagnosed with pituitary CCA based on the surgical pathology and immunohistochemistry findings. Both patients had residual visual defects on follow-up.

CCAs are a rare clinicopathological subtype of pituitary adenomas. An extensive review of 81 cases published in the English literature showed that CCAs are more common in women (72.8%) and have a mean age occurrence of 47.8 years [3]. Unlike the mean age of CCAs, one of the presented cases was considerably older at 84 years. Clinically, CCAs are typically invasive macroadenomas. Existing reports suggest that most cases (65%) with CCA present with Cushing syndrome [3]. Both cases presented with acute visual symptoms and did not exhibit signs or symptoms of Cushing syndrome. Like the presented cases, existing reports demonstrate that cases typically present with visual dysfunction and headache (33.7% and 29%, respectively) [3]. Given that both cases exhibited no symptoms relatively late in their disease course, treatment was initiated only after both cases presented to the emergency department with visual complaints. Given the high acuity of their symptoms and large size of both of their tumors, both casesunderwent urgent surgery and so dynamic pituitary testing for Cushing syndrome was not performed.

Our first case received radiotherapy because of invasion into the sphenoid sinus, whereas the second case was not offered radiotherapy given her age and the prohibitive side effects of memory and cognitive changes that could accompany radiation to the brain. Due to the rarity of CCA, there are currently no guidelines on treatment, monitoring, and prognosis. The current treatment of CCAs is surgical resection with or without adjuvant radiation treatment, accompanied by annual brain imaging [3]. CCA can transform to Crooke cell carcinoma and can cause drop dural metastases within the spinal canal, as well as systemic metastasis, although this is unusual. According to a recent comprehensive case series, 9.7% of CCAs demonstrated systemic metastasis [3]. Furthermore, CCAs have a low success rate at reoperation and exhibit recurrence rates as high as 60% [4]. Tumors that display invasion, postoperative residual, or postoperative recurrence may require adjuvant Gamma Knife radiotherapy [5]. A recent study demonstrated that 40% of cases who received radiotherapy had a 30% reduction in tumor volume and had no tumor recurrence or growth during the 12-month follow-up [6]. Adverse effects of Gamma Knife radiotherapy include hypopituitarism in 20% to 40% of cases, optic neuropathy, seizures, memory loss, and hemiparesis [7]. The adverse effects from radiotherapy are more likely in cases who present with large tumors with peritumoral edema and require high-dose radiation [8]. Notably, temozolomide, an oral alkylating agent, can be effective for treating refractory pituitary adenomas and carcinomas [3].

CCAs are immunopositive for the transcription factor T-PIT, distinguishing corticotroph adenomas from other pituitary adenoma types [1]. A small subset of corticotroph adenomas will have accumulation of paranuclear keratin filaments in the presence of excess glucocorticoids that give the cells a distinct pale, eosinophilic, hyalinized appearance of the cytoplasm, termed *Crooke hyaline* [4]. Cytokeratin immunostains can highlight the Crooke hyaline in a concentric pattern. The presence of the hyaline displaces the ACTH granules to the periphery of the cell and results in variable ACTH immunoreactivity that is negative around the nucleus [4]. While Crooke hyaline can be identified in scattered cells of many corticotroph adenomas, the presence of the material in more than 50% of cells, a key diagnostic criterion for CCA, is significant and suggests a more aggressive clinical behavior.

Despite the dismal recurrence rate of CCAs, both cases achieved successful remission after 4 and 2 years of follow-up respectively, but they will need to be monitored closely with annual brain imaging and eye exam. Given the delicate anatomy of the sella, and potential for post surgical complications, a multidisciplinary team of endocrinology, neuroradiology, neuropathology, neurosurgery, neuro-ophthalmology, and otolaryngology were required to manage the presented cases. Cases with CCAs are best managed in tertiary institutions, where the various specialties necessary to provide care for their multiple, complex presentations are readily available. In conclusion, even in the absence of Cushing syndrome, CCA should always be considered given its aggressive nature and close follow-up required.

## Learning Points

- CCA is a rare and aggressive corticotroph-derived adenoma that may be clinically nonfunctional or secrete ACTH resulting in subclinical or clinical Cushing syndrome.
- CCAs are defined by the presence of more than 50% of the tumor cells containing Crooke hyaline.
- Treatment of CCAs is usually surgical resection with or without adjuvant radiation therapy and/or temozolomide for recurrent cases. Lifelong head imaging surveillance and eye exam for recurrence is recommended.

## Data Availability

Data sharing is not applicable to this article as no data sets were generated or analyzed for this case report.

## Abbreviations

ACTH: adrenocorticotropin
CCA: Crooke cell adenoma
CT: computed tomography
MRI: magnetic resonance imaging
RR: reference range
T-PIT: T-box transcription factor
